# Better Ce (III) Sorption Properties of Unprocessed Chitinous Waste from *Hermetia illucens* than Commercial Chitosans

**DOI:** 10.3390/ma17215255

**Published:** 2024-10-29

**Authors:** Justyna Bąk, Piotr Bulak, Monika Kaczor, Dorota Kołodyńska, Andrzej Bieganowski

**Affiliations:** 1 Department of Inorganic Chemistry, Institute of Chemical Sciences, Faculty of Chemistry, Maria Curie-Skłodowska University, Maria Curie-Skłodowska Sq. 2, 20-031 Lublin, Poland; justyna.bak@mail.umcs.pl (J.B.); dorota.kolodynska@mail.umcs.pl (D.K.); 2 Institute of Agrophysics, Polish Academy of Sciences, Doświadczalna 4, 20-290 Lublin, Poland; m.kaczor@ipan.lublin.pl (M.K.); a.bieganowski@ipan.lublin.pl (A.B.)

**Keywords:** black soldier fly, chitin, waste biomass, cerium, biosorbent, rare earth elements, *Hermetia illucens*, waste

## Abstract

Insect farming generates a new type of chitinous waste in the form of dead specimens that have died of natural causes and insect moults (puparia), particularly large amounts of which are generated during the rearing of holometabolous insects. Following the circular economy paradigm, we treated waste in the form of puparia and dead adults of *H. illucens* as a valuable material, i.e., as sources of chitin, and tested it as a sorbent for cerium, a lanthanide of great industrial importance. For comparison, non-treated, raw insect materials and commercial chitosans were also investigated. Chitin extracted from *H. illucens* showed an adsorption capacity at the same level as commercially available, marine-source chitin (approximately 6 mg Ce·g^−1^). However, more interestingly, raw materials exhibited much higher adsorption capacities—dead adults were similar to commercial chitosans (approximately 32 mg Ce·g^−1^), while puparia demonstrated twice the performance (approximately 60 mg Ce·g^−1^). This indicates that unprocessed waste can be used as environmentally friendly, cost-effective Ce biosorbents with comparable or even better sorption capacity than chitosans, whose production requires intense chemical processing.

## 1. Introduction

Rare earth elements (REE) are represented by all the elements of the lanthanide group, as well as scandium and yttrium [1]. These elements are characterized by having similar physical and chemical properties, and their potential in technological applications depends on their unique catalytic [2], magnetic [3] and optical [4] properties. Their use in various technological processes is highly indispensable. REE are applicable in areas such as electronics and various medical fields, as well as manufacturing, technology, and renewable energy [5].

One of the most mentioned lanthanides is cerium. Its abundance in the earth’s crust is approximately 63 mg·kg^−1^ [6]. In industrial applications, it is most commonly used as a catalyst, e.g., in chemical processes, automotive technologies or petroleum refining, in polishing techniques, or as a luminophore in electronic devices [7]. However, it is also utilized in biomedical applications in the form of antimicrobial nanocompounds [8], and it may have potential usage in hydrogen production [9].

The consequence of the wide use of cerium in industry is the need to recover it at the end of the life cycle of the products in which it is found. This is important for both environmental and economic reasons. The extraction of cerium, whether from ore or through its recovery from end-of-life products containing it, mainly involves the phenomenon of sorption. This is because this procedure can be highly selective and can also process low-concentration sources. A broad variety of substances, including biosorbents, carbon-based supports and inorganic sorbents, as well as chelating and ionic-exchange resins, have been evaluated for their use in the sorption of cerium and REE [10].

Biosorbents are particularly interesting materials because they usually do not require pre-processing, or when they do it is quite simple to carry out. The basis of biosorption depends on the ability to bind various compounds (e.g., metals) on the surface of a given material (biosorbent) [11]. In the case of Ce sorption, there are a number of studies on different types of biosorbents. Among them, we can find methacrylic acid-grafted chitin with a sorption capacity of 147 mg Ce/g [12], marine chitosan and its analogues (29.45–119.48 mg Ce/g) [13], thiosalicylic-modified/ion-imprinted chitosan (164 mg Ce/g) [14], active carbons modified by KMnO4 (48–71 mg Ce/g) [15]. The given examples of biosorbents, despite their high sorption capacity, require operating costs and are not neutral to the environment due to the use of various chemicals for their production. More environmentally friendly examples of biosorbents include plant-based biosorbents, e.g., *Platanus orientalis* leaf powder, which showed a sorption capacity of 32.05 mg/g Ce [16], bacterial biomass of Spirulina (38.2 mg Ce/g) [17], and algal biosorbents of *Turbinaria conoides* (152.9 mg Ce/g) [18]. In the case of these examples, sorption occurred on unprocessed materials, but their biomass was still produced specifically for these purposes. This indicates the need to find biomaterials with already high sorption properties for, e.g., the removal of heavy metals or precious elements, such as REE. The search for biosorbents with a high capacity for Ce should align with the circular economy paradigm, which is based on the utilization of waste materials from one industrial branch as a source substrate for others, or its direct usage in new contexts. An example of such an approach can be the use of waste in the form of crab shell particles, which had a sorption capacity of 144.9 mg Ce/g, but this material needed acid pretreatment to remove excess calcium carbonate contained in it [19]. The less processed/modified the biosorbent is, the better it is in terms of costs and environmental aspects (no energy inputs and no consumption of chemical reagents).

In recent years, the market for the production of insects has been developing quite intensively. Their relatively simple breeding process allows for obtaining a high-protein product used in the feed industry [20]. One example of such insects is *Hermetia illucens*, which belongs to the family Stratiomyidae from the Diptera order [21]. This fly represents one of the holometabolous insects, which are characterized by a developmental cycle that passes through the pupa stage. This is followed by the adult form (imago), which leaves puparia (empty pupae molts) after hatching [22]. This kind of insect waste has various applications, including as a substrate for biochar production [23] or material for insect chitin extraction [24]. Increased interest in insect production will result in larger amounts of post-breeding residues as well as chitinous insect wastes, like puparia or dead adults after the end of their life cycle, which should be managed while taking into account the environmental policy of a circular economy.

Therefore, the main aim of this research was to examine the sorption properties of *H. illucens* post-breading remains (in the form of the puparia and dead adults of the insect) in relation to cerium ions. We hypothesized that such unprocessed material would be a good cerium sorbent. However, due to the fact that chitin sorbents obtained from other organisms are available on the market, an additional aim of this work was to check the sorption capabilities of pure chitin isolated from breeding pristine wastes—also from puparia and dead adults of the insect. The hypothesis underlying the implementation of the additional aim was the assumption that cerium sorption on chitin from *H. illucens* would be at least as effective as on commercially available chitin from marine origins.

## 2. Materials and Methods

### 2.1. Insect Breeding

*H. illucens* larvae were reared in the laboratory of the Institute of Agrophysics of the Polish Academy of Sciences in Lublin (Poland). The larvae were fed with commercial carp fish feed (FloraZoo, Chełmża, Poland) with the following composition (values given per dry weight): 54.80% carbohydrates, 25.00% protein, 5.00% fat and oil, 5.80% crude fiber, 5.70% ash, 1.25% lysine, 1.00% calcium, 0.97% phosphorus and 0.40% methionine. The culture temperature was 27 ± 1 °C with a substrate humidity of 50%–80% [25]. The insects were kept in darkness until the pupae occurred after 16 days. They were then collected and transferred to the breeding container where the adults emerged (photoperiod 12/12, air relative humidity 60%, 25 ± 1 °C). The empty puparia were collected for further processing at this stage. Under these conditions, the adult flies reproduced and lived for up to five days. During this time, they were provided with access to water. After mating, the adults died of natural causes and were removed from the chamber.

### 2.2. Chitin Extraction

**Figure 1:**
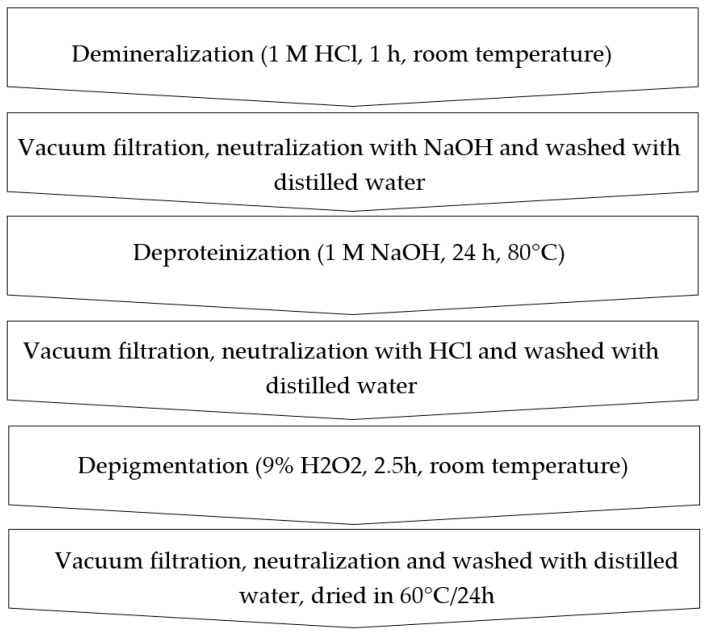
Scheme of chitin extraction from *H. illucens* puparia and dead flies.

Sodium hydroxide, hydrochloric acid, ethanol and hydrogen peroxide (30%) were acquired from Standard (Lublin, Poland). The empty puparia and dead flies were cleaned utilizing ethanol and water, then dried at 60 °C for 24 h and ground. Size distribution can be found in Table S1 (in the Supplementary Materials). Chitin extraction was then performed (Figure 1) according to Złotko et al. [26].

### 2.3. Sorbents

The following materials have been used for the experiments assessing the adsorption of Ce(III) ions: commercial chitin, chitin obtained from *H. illucens* adults (flies)—Ch-A, chitin derived from *H. illucens* puparia—Ch-P, the ground form of *H. illucens* adults—A, the ground form of *H. illucens* puparia—P, high-molecular-weight commercial chitosan—CS-HW, medium-molecular-weight commercial chitosan—CS-MW, and low-molecular-weight commercial chitosan—CS-LW.

Commercial chitin derived from shrimp shells was purchased from Sigma Aldrich (Burlington, MA, USA) (chemical formula: (C_8_H_13_NO_5_)n, powder form). In addition, chitosan samples of different molecular weights (CS-HW, CS-MW and CS-LW) acquired from Sigma Aldrich (Burlington, MA, USA) were used to compare the efficiency of the removal of cerium(III) ions from aqueous solutions. All types of chitosan have the chemical formula C_12_H_24_ClN_2_O_9_ and occur in a powder form.

Prior to analysis, samples of Ch-A, Ch-P, A and P were ground with an IKA laboratory mill (Tube Mill Control, DanLab, Białystok, Poland). The characterization of the particle distribution of the obtained powders was carried out with an automated particle classification system, Morphology G3 (Malvern Panalytical, Malvern, Great Britain). Particle size distributions are given in Table S1.

### 2.4. Physicochemical Characterization

The materials underwent X-ray diffraction (XRD) characterization and infrared spectroscopy attenuated total reflectance Fourier-transform infrared (ATR-FTIR) analysis was conducted before the sorption process. Measurements of N_2_ adsorption/desorption isotherms were carried out using the accelerated surface area and porosimetry system (ASAP) method before sorption. Subsequently, scanning electron microscopy (SEM), coupled with energy-dispersive X-ray spectroscopy (EDX), was employed to examine the materials after the sorption process.

For the X-ray diffraction analysis, the mineral composition was determined using the powder method with the Panalytical X’Pert PROMPD X-ray diffractometer, which featured the PW 3050/60 goniometer and operated in the 2θ angle range of 5–65 degrees (Malvern Panalytical, Malvern, Great Britain). The X-ray source utilized was a copper lamp (CuK = 0.154178 nm). The ground, well-distributed sample was placed in a special XRD holder, which is made of a material that does not reflect X-rays. The basis of the XRD method is the phenomenon of X-ray diffraction on the crystal lattice of a substance. Data analysis was performed with the X’Pert High Score software.

In the case of ATR-FTIR spectroscopy, the spectra of the eight sorbents were recorded before the sorption using the ATR technique. This analysis was conducted within the wavelength range of 550–4000 cm^−1^, employing the Agilent Cary 630 FTIR spectrometer (Agilent Technologies, Santa Clara, CA, USA). ATR-FTIR spectra were performed by placing a sample of about 0.2 g directly on a diamond crystal and pressing the sample against the crystal to ensure adequate contact. The spectroscope then collects the data without further sample processing. Spectral data were recorded with the Micro Lab PC program, and a subsequent evaluation of the spectra was carried out with the Agilent Resolutions Pro software (Agilent Technologies, Santa Clara, CA, USA).

An ASAP 2420M (Micromeritics Instrument Corporation, Norcross, GA, USA) sorption analyzer was used to calculate the low-temperature N_2_ adsorption/desorption isotherms. The materials (0.1 g) were degassed at 105 °C in a glass tube before the measurements were taken for removing volatile components of the samples. Both the surface area and the pore size distribution were calculated with the Barrett-Joyner-Halenda (BJH) and Brunauer-Emmett-Teller (BET) methods, respectively.

The Quanta 250 FEG scanning electron microscope (SEM) (FEI, Hillsboro, OR, USA) equipped with energy-dispersive X-ray spectroscopy (EDX) was used to study the microstructural morphology and elemental composition following the sorption process. Before the surface morphology was measured, a sample was placed on a special pad, sputtered with a carbon-conductive material, and then placed in the chamber of the microscope.

### 2.5. Batch Sorption Test

To evaluate the effectiveness of the sorption process, batch sorption tests were conducted involving the removal of Ce(III) ions using eight biopolymers from water media. As a source of cerium(III) ions, cerium nitrate hexahydrate (Avantor Performance Materials, Gliwice, Poland) was utilized at a concentration of 1000 mg·L^−1^. First, the effect of the solution pH (2.0–6.0) on the sorption process was tested by weighing 0.04 g of sorbents (commercial chitin, CS-HW, CS-MW, CS-LW) and shaking in a solution of cerium(III) ions (20 mL) with an initial concentration of 50 mg·L^−1^ for 300 min. Studies of the optimal pH selection (pHM82, Radiometer, Copenhagen, Denmark) were performed for the commercial biopolymers due to the limited amount of remains. Then, after applying the same starting concentration of 50 mg·L^−1^, the kinetic investigation was carried out at various adsorption times (0–300 min). In addition, evaluations of the effect of the initial concentration (10–200 mg·L^−1^) on the sorption process (isotherm studies) were also performed at a constant shaking time of 300 min.

In summary, shaking was carried out at various times (0–300 min) and initial concentrations (10–200 mg·L^−1^) at a pH of 3.0 (optimum pH) and a temperature of 25 °C, at a rate of 180 rpm. A laboratory shaker (type 458A, Elpin Plus, Lubawa, Poland) was used for static tests. Nitric acid and/or sodium hydroxide (Chempur, Piekary Śląskie, Poland) solutions in small amounts were added to adjust the pH, which was measured by a pHM82 pH meter (Radiometer, Copenhagen, Denmark). Each time, the suspension was immediately filtered after the specified time period and the concentration of cerium(III) ions was detected by inductively coupled plasma optical emission spectroscopy (ICP-OES, type 720, Varian, Palo Alto, CA, USA). The concentration of Ce(III) ions was determined with the following parameters: wavelength of 446.021 nm, resolution of 0.004 nm, reading time of 10 s, sampling delay of 15 s, pump speed of 15 s and rinse time in the range of 12–15 s. Filtration was performed using quantitative-medium sieves to separate the sorbent from the solution, which made it possible to determine the concentration of Ce(III) ions in the solution after the sorption process. Errors were noted during the triplicate testing and are represented in the figures as 5% error margins.

### 2.6. Analytical Methods

This subsection presents (in the Supplementary Materials) the formulas used to calculate the amounts of adsorbed ions, equilibrium capacities, and nonlinear forms of the kinetic and isotherm models, as well as the correlation coefficient and Chi-square error.

## 3. Results and Discussion

### 3.1. The Change in the Physicochemical Properties of Biopolymers

#### 3.1.1. Mineral Composition

**Figure 2:**
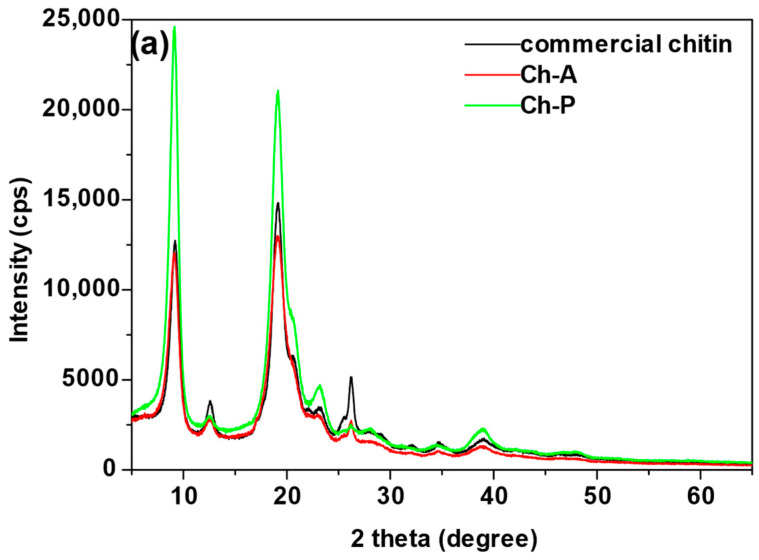

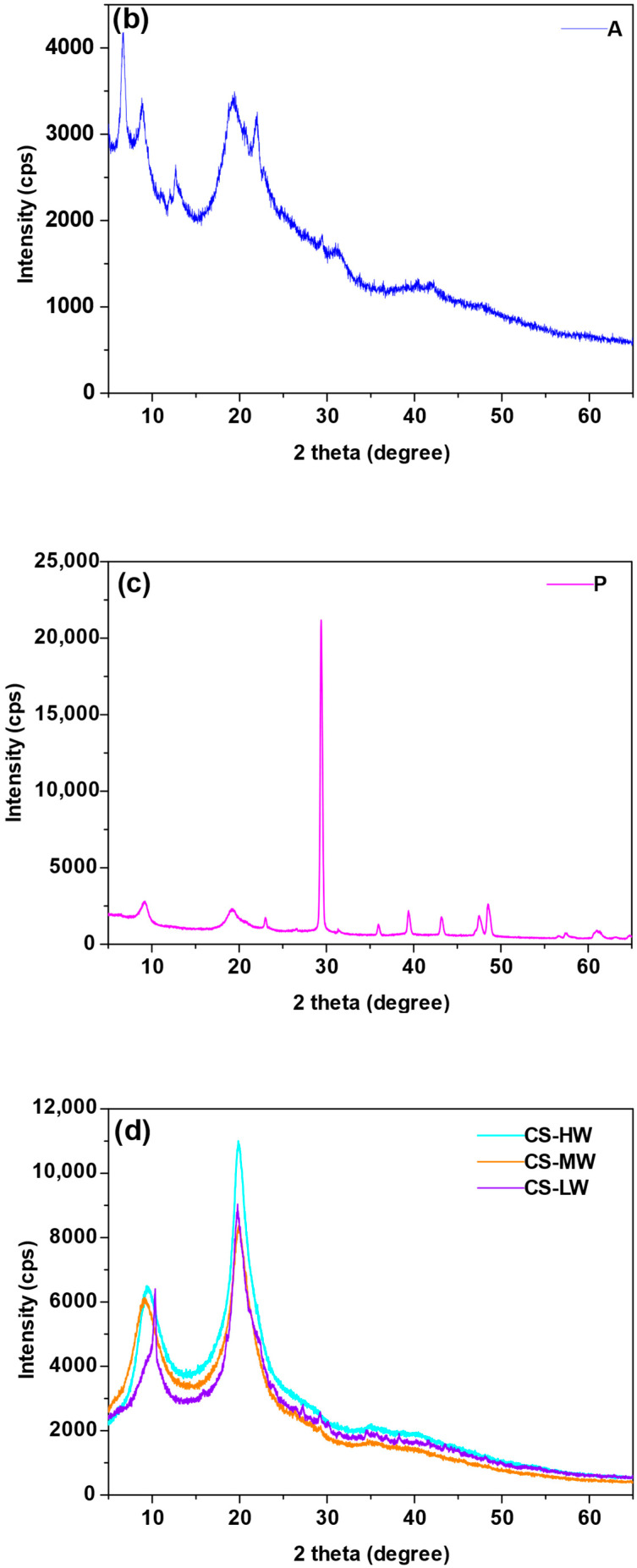
X-ray diffractometry analysis of (**a**) commercial chitin, chitin obtained from *H. illucens* adults (Ch-A), chitin derived from *H. illucens* puparia (Ch-P), (**b**) the ground form of *H. illucens* adults (A), (**c**) the ground form of *H. illucens* puparia (P), (**d**) high-molecular-weight commercial chitosan (CS-HW), medium-molecular-weight commercial chitosan (CS-MW), and low-molecular-weight commercial chitosan (CS-LW).

The mineral composition of the eight sorbents, as determined by X-ray diffraction (XRD), is shown in Figure 2.

In the case of commercial chitin, the primary peak occurs at 2θ = 19.2. This is a diffraction peak corresponding to the N-glucosamine in the chain, while the secondary peak at 2θ = 9.1 is smaller and is due to the presence of N-acetyl-D-glucosamine [27]. The existence of these peaks indicates the crystalline structure of chitin, which can occur in three forms: α, β or γ [28]. Additional peaks at 2θ angles of 26.2 and 38.9 are characteristic of silica. The similarity of the spectra of chitin samples (Ch-A and Ch-P) to the XRD spectrum of commercial chitin testifies to the efficiency of chitin synthesis based on these waste materials. In the case of Ch-A and Ch-P, there are two characteristic diffraction peaks in the spectrum with the highest intensities at 2θ angles of 9.1 and 19.1. These results agree with those obtained by Waśko et al. [29], who characterized chitin obtained from the puparium of *H. illucens* and determined its α form of crystallinity. Other peaks with low intensities at 2θ angles 26.2, 34.6 and 38.9 are characteristic of silica.

In the case of the spectrum of the sample derived from grinding the adults (A), a number of peaks appear, and two of them at 2θ angles 22.0 and 31.9 are matched by calcium and aluminum silicate. This spectrum shows a raised background, indicating the presence of amorphous substances in this sample. As for the spectrum of the sample attained by grinding *H. illucens* fly puparia (P), peaks matching calcium carbonate were obtained at the following 2θ angles: 23.0, 29.3, 35.9, 39.4, 43.3, 47.5 and 48.5. For all the chitosan samples (CS-HW, CS-MW, CS-LW), two characteristic broad peaks are observed in the X-ray diffraction spectrum. The primary peak of chitosan corresponds to -NH_2_ groups in amine II and occurs at 2θ equal to 19.9. At 2θ equal to 9.4 there is a secondary peak. This is a less intense diffraction peak, which is related to the existence of -N-CO-CH_3_ groups in amine I contained in the structure of chitosan [30].

#### 3.1.2. The Presence of Functional Groups

**Figure 3:**
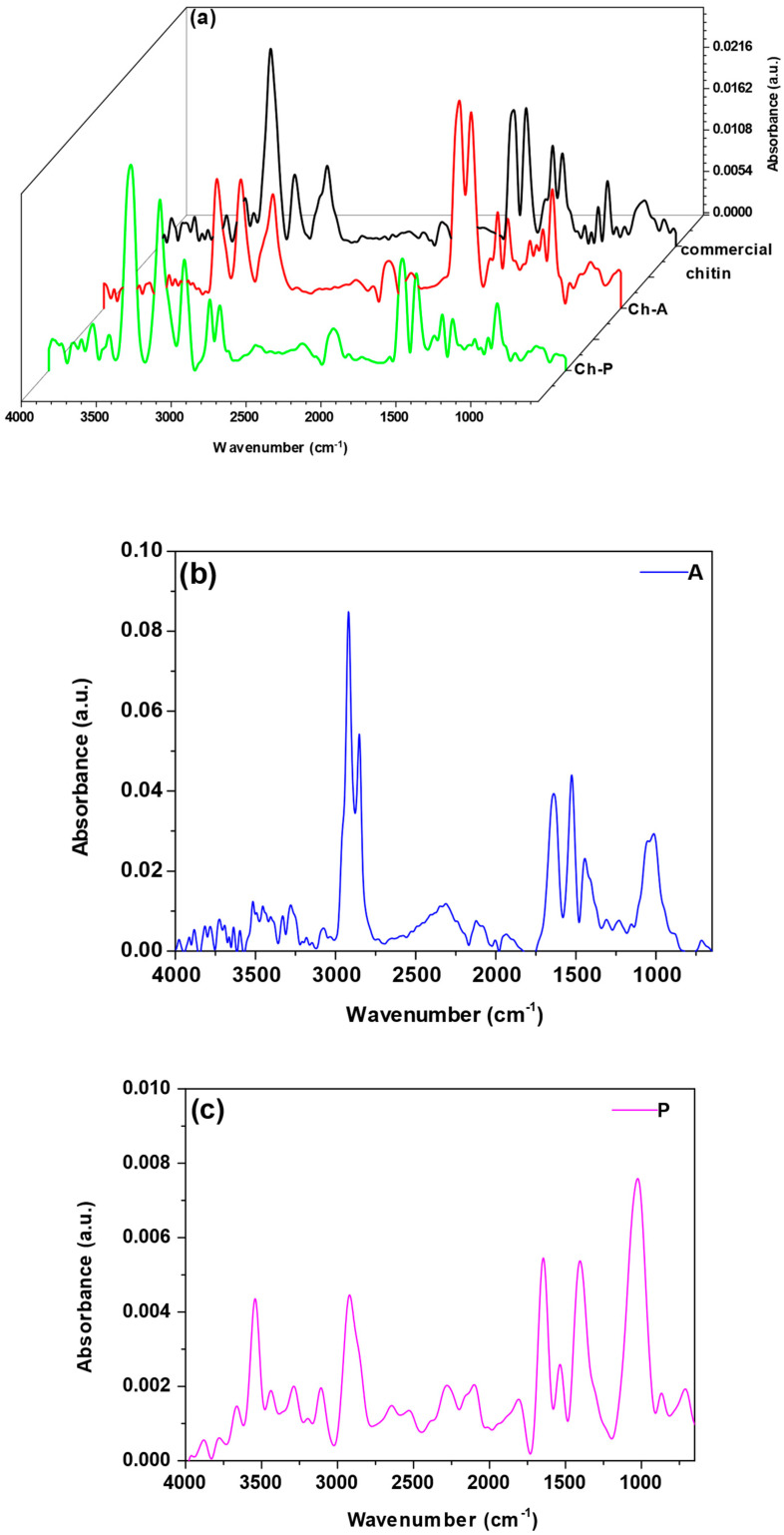

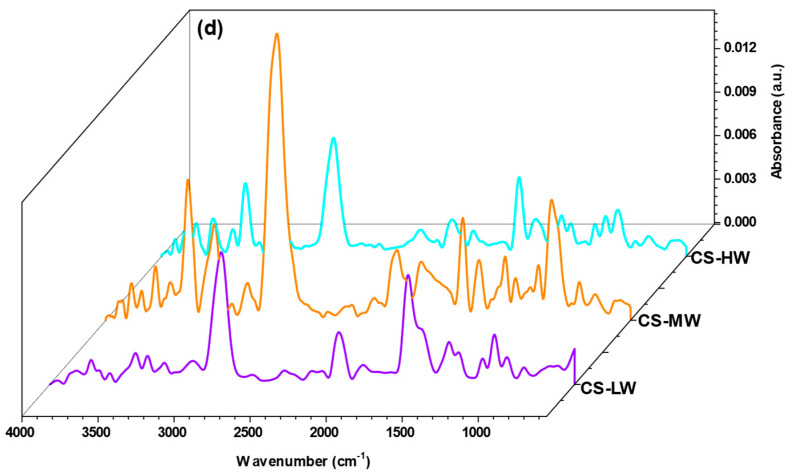
ATR-FTIR method of (**a**) commercial chitin, chitin obtained from *H. illucens* adults (Ch-A), chitin derived from *H. illucens* puparia (Ch-P), (**b**) the ground form of *H. illucens* adults (A), (**c**) the ground form of *H. illucens* puparia (P), (**d**) high-molecular-weight commercial chitosan (CS-HW), medium-molecular-weight commercial chitosan (CS-MW), and low-molecular-weight commercial chitosan (CS-LW).

Based on ATR-FTIR analysis (Figure 3), it is possible to indicate the characteristic surface functional groups of the materials. For all the sorbents, the peaks in the range of 3800–3400 cm^−1^ and 3350–3200 cm^−1^ correspond to O-H and N-H bonding stretching vibrations, respectively [31,32]. In the case of sorbent A, these peaks have the lowest intensities. High-intensity peaks at approximately 2920–2850 cm^−1^ are considered responsible for the C-H stretching vibration in aliphatic chains [33]. In some cases, such as in the Ch-P and A samples, there are two peaks associated with these vibrations. The existence of a peak at approximately 1640 cm^−1^ (stretching of C=O groups in amide groups, amide I band) has been used to confirm the presence of residual N-acetyl groups. The amide II band (1550 cm^−1^) corresponds to the bending vibrations of N-H groups in amide [34,35,36].

In the case of the chitosans (CS-HW, CS-MW, CS-LW), the intensity of the peaks at 1640 and 1550 cm^−1^ is lower than that of the commercial chitin, Ch-A and Ch-P samples. This difference can be attributed to the deacetylation reaction, during which some of the acetamide groups of chitin are converted to amino groups [37,38]. In the A, P and CS-HW samples, the peaks appear at approximately 1420 cm^−1^, originating from the bending vibrations of CH_2_ groups. In some sorbents, including the commercial chitin, Ch-A, Ch-P, CS-HW, CS-MW and CS-LW samples, peaks emerge at approximately 1370 cm^−1^, linked to the presence of deformation vibrations from CH_3_ groups. With the exception of sample P, the amide III band (1310 cm^−1^) indicates the existence of C-N groups in the sorbents [34,35]. These peaks are characterized by less intense absorbance. In addition, C-O-C vibrations are associated with the band between 1070 and 1000 cm^−1^ [39,40]. The occurrence of C-N and C-C groups is further evidenced by small peaks that become visible at approximately 870 cm^−1^ [41].

#### 3.1.3. Porosity Characteristics

Based on the gas porosimetry method, the specific surface area, pore diameter and pore volume of the eight materials have been determined. The results are shown in Table 1.

The gas adsorption porosimetry results reveal significant variations in the surface area and pore size distribution of the tested biopolymers. Commercial chitin exhibits the highest specific surface area at 5.28 m^2^·g^−1^, indicating that there is a relatively high surface area available for adsorption processes. In contrast, Ch-P and P have the lowest specific surface areas of 0.11 m^2^·g^−1^ and 0.03 m^2^·g^−1^, respectively, suggesting that they have fewer exposed surface areas for adsorption. Of the chitosan groups, CS-MW has the largest specific surface area of 1.21 m^2^·g^−1^. Regarding the pore diameter, P has the largest pores, with an average diameter of 41.4 nm, followed by CS-LW with a pore diameter of 34.9 nm. Ch-A has the smallest pores, with an average diameter of 7.7 nm. From these data, it is possible to determine which pore sizes dominate in each material. Mesopores and even macropores are dominant in sample P. In addition, a predominance of mesopores is found in the samples of commercial chitin, Ch-P, A, CS-HW, CS-MW and CS-LW. In contrast, mesopores and micropores are present in the Ch-A sample. Pore diameter is an essential parameter as it can influence the types of molecules that can be adsorbed. In addition, the pore volume was also determined. Among the tested commercial sorbents, the highest pore volume was obtained for chitin 0.0238 cm^3^ g^−1^. From the group of Ch-A, Ch-P, A and P sorbents, the largest pore volume was obtained for Ch-A 0.0068 cm^3^ g^−1^.

#### 3.1.4. Morphology and Elemental Composition

**Figure 4:**
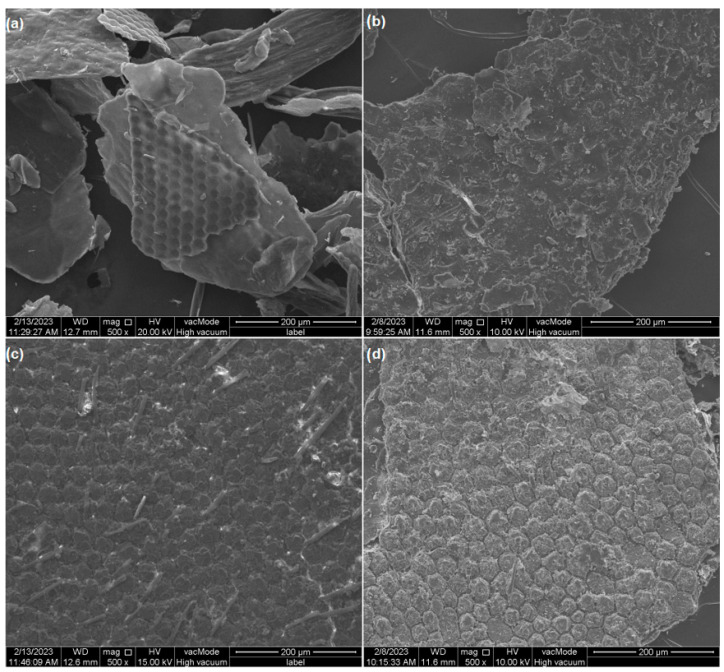

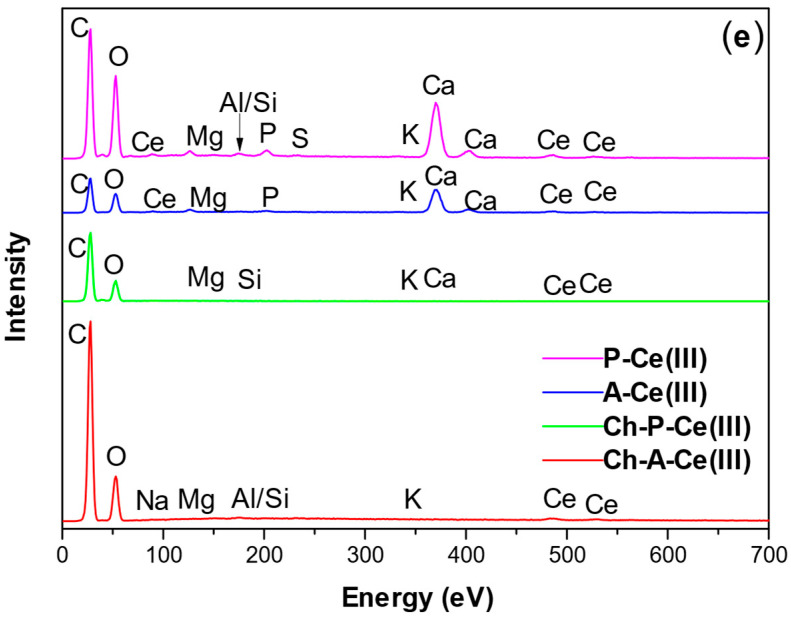
Morphology of the samples under 500x magnification in SEM: (**a**) chitin obtained from *H. illucens* adults (Ch-A), (**b**) chitin derived from *H. illucens* puparia (Ch-P), (**c**) the ground form of *H. illucens* adults (A), (**d**) the ground form of *H. illucens* puparia (P), (**e**) EDS patterns for Ch-A, Ch-P, A, P after Ce sorption.

SEM analysis shows the surface shape of the isolated chitin particles as well as the starting materials (Figure 4a–d). The surface of the chitins is much smoother than that of raw adults and puparia. The chitin from adults is particularly smooth, and in the foreground, the exoskeleton covering the insect’s compound eye can likely be observed. Individual cavities in this fragment correspond to the single ommatidia. Chitin from puparia (Ch-P) has a surface composed of superimposed plates dotted with numerous pits.

The ground exoskeletons of adults and puparia exhibit a clearly visible structure approximately resembling a honeycomb. The description of this type of surface also appears in other publications characterizing the microstructure of *H. illucens* [23,24,42]. In the case of the adults, numerous sensory hairs are visible, while the puparium surface in the captured fragment contains a much smaller number of them.

Samples of investigated materials after Ce(III) adsorption were also analyzed through energy-dispersive spectroscopy (Figure 4e). EDS spectra for commercial chitin and chitosans are presented in Figure S1 (in the Supplementary Materials). All the materials show the presence of Ce. In the samples of Ch-A and Ch-P, the occurrence of Si can be connected to silica identified by XRD in both of the samples. In A and P, a peak for Ca is clearly visible, which is not seen in the Ch-P and Ch-A samples, and which can be linked to the incidence of CaCO_3_ in the *H. illucens* exoskeleton [43]. This compound has also been detected by XRD analysis in the P sample. The occurrence of CaCO_3_ may influence the higher sorption capacity of Ce (III) ions by A and P compared to their chitin isolates. The presence of this compound may lead to carbonate ions-mediated microprecipitation process and subsequent deposition on the “rough” surface of these materials (Figure 3a,b) [19]. Other elements like Mg, Al, Na, Si, P, S, and K, detected in minor qualities in the samples, occur naturally in *H. illucens* in significant quantities [44]. In research publications it can be found that demineralization of the sample during chitin extraction is never complete, which is why the EDS spectra for Ch-P and Ch-A also contain mineral peak (Figure 3). For example, Hahn et al. [45] also using hydrochloric acid obtained mineral removal of approximately 90% for puparia. For other types of reagents, e.g., formic acid, demineralization of 85% was achieved for puparia and 87% for flies [46]. However, higher efficiencies can be obtained with natural deep eutectic solvents of approximately 98% [47].

### 3.2. Cerium(III) Ions Sorption Characteristics

#### 3.2.1. pH Test

**Figure 5:**
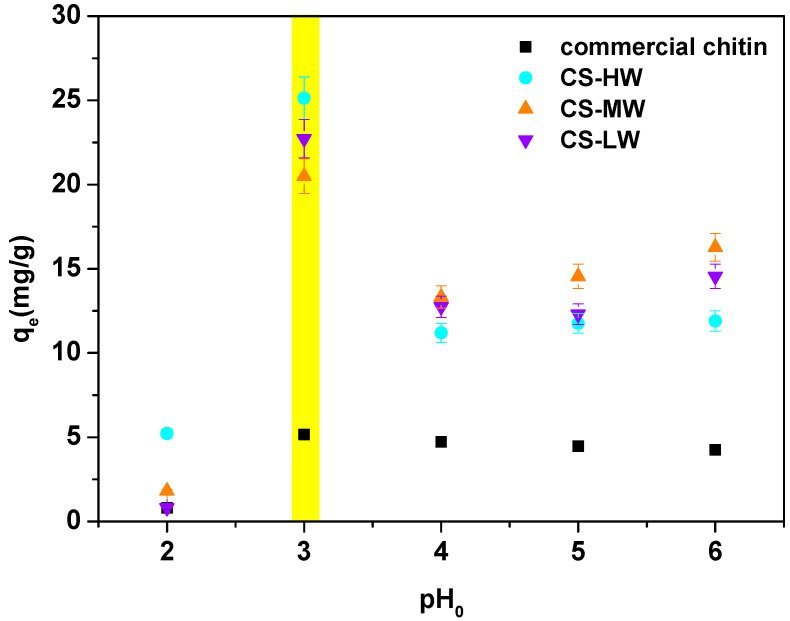
pH effect for the Ce(III) ions sorption on commercial chitin, high-molecular-weight commercial chitosan (CS-HW), medium-molecular-weight commercial chitosan (CS-MW), and low-molecular-weight commercial chitosan (CS-LW) (C0 50 mg·L^−1^, t 300 min., T 25 °C, 180 rpm). Yellow line indicated the most interesting result.

In general, pH is regarded as a significant factor that regulates the processes of adsorption at water-adsorbent interfaces. The impact of the initial solution pH (Figure 5) was investigated in order to establish the ideal conditions for the cerium(III) ions sorption on biopolymers. Due to the limited number of Ch-A, Ch-P, A and P samples, studies of the effect of pH were conducted only on commercially purchased materials.

Figure 5 shows that the sorption efficiency has a strong dependence on the solution pH. The optimum is found at a pH of 3.0 and the q_e_ values are equal to 5.16 mg·g^−1^ for commercial chitin, 25.13 mg·g^−1^ for CS-HW, 20.50 mg·g^−1^ for CS-MW and 22.72 mg·g^−1^ for CS-LW. At this pH value, the amine groups are protonated to -NH_3_^+^ and the hydroxyl ones to -OH_2_^+^. In the case of amide groups, protonation occurs through oxygen [48]. At a pH in the range of 4.0 to 6.0, a significant reduction in the amount of adsorbed Ce(III) ions is observed, which may be due to cerium(III) hydroxide precipitation at higher pH values [49]. On the other hand, the low sorption capacity at low pH 2 may be due to competition of Ce(III) ions with H_3_O^+^ ions [50]. The significant difference between the sorption properties of commercial chitin and chitosans (Figure 4), may be explained by the higher crystallinity of chitin than chitosan. In turn, crystallinity may affect the availability of reactive functional groups by reducing the level of hydration of the biopolymer molecule [51].

#### 3.2.2. Sorption Time Effect and Kinetic Fitting

**Figure 6:**
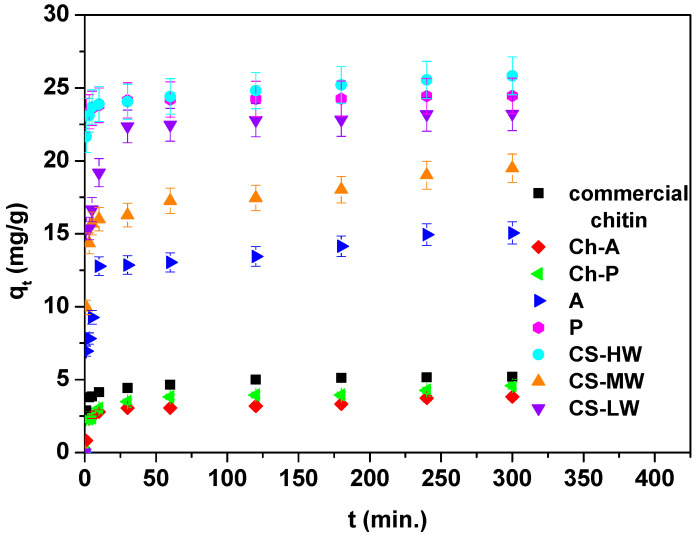
Contact time effect for the Ce(III) ions sorption on commercial chitin, chitin obtained from *H. illucens* adults (Ch-A), chitin derived from *H. illucens* puparia (Ch-P), the ground form of *H. illucens* adults (A), the ground form of *H. illucens* puparia (P), high-molecular-weight commercial chitosan (CS-HW), medium-molecular-weight commercial chitosan (CS-MW), and low-molecular-weight commercial chitosan (CS-LW) (C0 50 mg·L^−1^, pH 3.0, t 1–300 min., T 25 °C, 180 rpm).

Another variable that has a significant impact on the speed of the sorption process is the phase contact time. Figure 6 shows the effect of phase contact time (1–300 min) of Ce(III) ions sorption on eight biopolymers at a concentration of 50 mg·L^−1^.

As can be seen in Figure 6, the kinetic diagram of the adsorption of Ce(III) ions consists of a fast phase at the beginning, where adsorption is rapid before it becomes constant after reaching equilibrium, indicating the exhaustion of access to sorption sites [52,53]. The initial stage’s quick adsorption of Ce(III) ions may have been facilitated by the larger concentration gradient and greater availability of free adsorption sites [54]. As is evident from Figure 6, commercial chitin, Ch-A and Ch-P reach equilibrium after 60 min. For A, P and all the chitosan samples, equilibrium is reached after 120–180 min. This is due to the fact that higher amounts of adsorbed Ce(III) ions were obtained for these materials. The q_t_ values achieved after a time of 300 min are 5.20, 3.82, 4.58, 15.06, 24.45, 25.83, 19.49 and 23.23 mg·g^−1^ for commercial chitin, Ch-A and Ch-P, A, P, CS-HW, CS-MW and CS-LW, respectively. In the chitin group, the best results have been attained for commercial chitin, but the q_t_ values observed for Ch-P are only slightly lower. These are immediately followed by sample P with a small q_t_ difference. In general, chitins show significantly lower sorption capacities for Ce(III) ions compared to chitosans. Satisfactory results have been acquired for the ground form of *H. illucens* adults. This last observation should be considered positive because it opens up the possibility of the potential use of *H. illucens* puparia and flies without requiring their conversion into chitin.

Based on a kinetic analysis, the rate and mechanism of the Ce(III) ions adsorption can be assessed. The experimental data have been correlated using three kinetic models in nonlinear forms: the pseudo-first-order (PFO), pseudo-second-order (PSO) and Elovich models. The parameters are listed in Table 2. The nonlinear fitting of kinetic models for Ce(III) ions sorption on biopolymers are presented in Figure S2 (in the Supplementary Materials).

The PFO model assumes that sorption occurs only at localized sites without interaction with other adsorbed ions. The PSO model, on the other hand, presumes that the amount adsorbed depends not only on time, but also on the concentration of the substance in solution. The third model used to describe the kinetics is the Elovich model. It infers that the activation energy increases as the sorption time increases, and the sorbent surface is considered to be heterogeneous. This model explains the occurrence of adsorption on localized sites and allows for interactions between adsorbed ions [55,56].

To identify the model that best describes the sorption process of Ce(III) ions, the values of determination coefficients and Chi-square errors are compared. The best fit occurs with the Elovich model, for which the highest R^2^ values and lowest errors were obtained. The exceptions are the Ch-A and CS-MW samples, for which a better fit is shown by the PSO model. In the case of sorbent W, there is a very good fit with all three kinetic models. This may indicate an interaction of a chemical nature between Ce(III) ions and biopolymers [57].

#### 3.2.3. Effect of Solution Concentration, Isotherm Fitting, and Sorption Comparison

**Figure 7:**
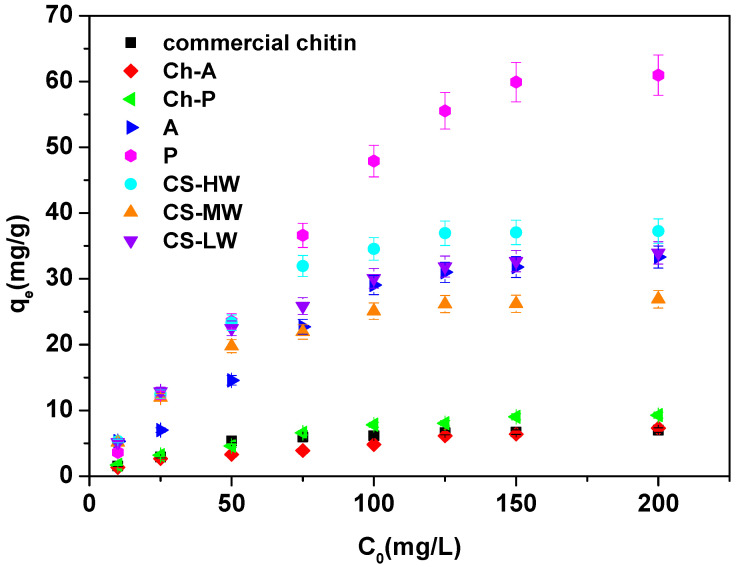
Initial concentration of solution effect for the Ce(III) ions sorption on commercial chitin, chitin obtained from *H. illucens* adults (Ch-A), chitin derived from *H. illucens* puparia (Ch-P), the ground form of *H. illucens* adults (A), the ground form of *H. illucens* puparia (P), high-molecular-weight commercial chitosan (CS-HW), medium-molecular-weight commercial chitosan (CS-MW), and low-molecular-weight commercial chitosan (CS-LW) (C0 10–200 mg·L^−1^, pH 3.0, t 300 min., T 25 °C, 180 rpm).

Figure 7 presents the effect in terms of Ce(III) ions sorption of the initial concentration of the solution (10–200 mg·L^−1^) on eight biopolymers at a time of 300 min.

Based on Figure 7, by increasing the initial concentration, the equilibrium capacity of Ce(III) ions increases. With a change in initial concentration from 10 to 200 mg·L^−1^, the qe values change as follows: 1.54–6.98 mg·g^−1^ for commercial chitin, 1.31–7.30 mg·g^−1^ for Ch-A, 1.71–9.27 mg·g^−1^ for Ch-P, 5.31–33.31 mg·g^−1^ for A, 3.61–60.96 mg·g^−1^ for P, 5.32–37.26 mg·g^−1^ for CS-HW, 5.13–26.90 mg·g^−1^ for CS-MW and 5.13–33.95 mg·g^−1^ for CS-LW. This is because the mass transfer is substantially higher, and the beads’ active adsorption sites saturate more quickly. The diffusion of the molecules into the solid is slower for a low starting concentration (10 mg·L^−1^) [58,59]. As in the case of the study of the contact time effect, it is chitin that adsorbs Ce(III) ions with the lowest efficiency. The chitosans and sample A are in second place. For higher concentrations, sample P shows the highest equilibrium capacities. This is an important observation that suggests that samples of only ground fly forms A and P retain their sorption properties in their natural state and do not require additional processing to achieve higher sorption efficiencies.

A total of three sorption isotherms were proposed to describe the Ce(III) ions sorption on biopolymers: the Langmuir, Freundlich and Temkin isotherms in nonlinear forms. The isotherm parameters are summarized in Table 3. The nonlinear fitting of the isotherm models for Ce(III) ions sorption on biopolymers is shown in Figure S3 (in the Supplementary Materials).

The Langmuir isotherm assumes that the adsorbate covers the adsorbent monolayer at specific sorption sites. Additionally, the adsorbate molecules are unable to migrate across the adsorbent’s surface and do not interact with one another [60]. On the other hand, the Freundlich model presupposes that there is multilayer adsorption on a heterogeneous surface as opposed to monolayer adsorption on a homogenous material surface [61]. Temkin’s model primarily considers the interactions between the adsorbent and adsorbate that take place during the sorption process. Additionally, it assumes that the heat of adsorption of adsorbed molecules linearly decreases as the sorption area’s coverage increases [62].

After observing the values of the determination coefficients calculated from the isotherms, the best fit to the Langmuir model can be found. This may indicate a homogeneous and flat adsorption surface, with equally valuable adsorption sites and no interactions between adsorbate molecules at adjacent sites. A good fit, moreover, occurs for the Temkin model. This may suggest some involvement of electrostatic interactions, such as between biopolymer surfaces and Ce(III) ions in the sorption process.

In the literature, a growing number of sorbents have been utilized to examine their sorption potential for the removal of cerium(III) ions from aqueous solution (Table 4). Comparing the equilibrium capacities obtained during the study, it can be seen that the results obtained for sample P are comparable to the results obtained using other sorbents. Importantly, this waste does not require processing and is itself a good biosorbent for cerium(III) ions, and its amount is increasing year by year. The use of unmodified waste does not require energy consumption, does not generate additional chemical waste associated with modification, and its use aligns with the principles of a closed-loop economy.

Summarizing our findings, the best adsorption of Ce(III) ions among the investigated samples obtained from *H. illucens* and the commercial materials occurred at pH 3. At a starting concentration of 50 mg Ce·L^−1^, all the chitins reached equilibrium faster (60 min) than the raw materials and chitosans (120–180 min). Under these conditions and after 300 min, the highest adsorption properties were expressed by high-molecular-weight commercial chitosan (25.83 mg·g^−1^), although the ground form of *H. illucens* puparia (24.45 mg·g^−1^) was very close to this level. The second raw material, the ground form of *H. illucens* adults (15.06 mg·g^−1^), was placed in an intermediate position between high-molecular-weight commercial chitosan and chitins. The weakest adsorption properties were shown by chitins and the difference between commercial ones and those from *H. illucens* was minimal (5.20 mg·g^−1^ commercial vs. 3.82 mg·g^−1^ for chitin obtained from *H. illucens* adult flies and 4.58 mg·g^−1^ for chitin derived from *H. illucens* puparia).

The effect of the initial Ce(III) concentration on its adsorption measured after 300 min was not considerable for all the chitins studied (the max qe values for the commercial chitin, chitin obtained from *H. illucens* adults flies, and chitin derived from *H. illucens* puparia were 6.98, 7.30, and 9.27 mg·g^−1^, respectively). The most interesting finding was that the material with the highest adsorption capacity was the ground form of *H. illucens* puparia (60.96 mg·g^−1^), while the ground form of *H. illucens* adults (33.31 mg·g^−1^) was placed at a level similar to all the commercial chitosans tested (37.26, 26.90, and 33.95 mg·g^−1^ for high-molecular-weight commercial chitosan, medium-molecular-weight commercial chitosan, and low-molecular-weight commercial chitosan—CS-LW, respectively). All the tested materials under these conditions had the best fit to the Langmuir model, with the exception of chitin obtained from *H. illucens* adults flies, where the best fit occurred for the Freundlich model.

## 4. Conclusions

The results of this research clearly showed that wastes from *H. illucens* breeding in the form of puparia and dead adults did not require processing and were themselves good biosorbents for cerium. Untreated chitinous waste had significantly better sorption properties for Ce(III) than the purified chitin extracted from them. However, chitins from *H. illucens* had slightly better adsorption properties than commercial marine chitin, but the difference was low. The use of unmodified waste does not require any energy inputs and does not cause additional chemical waste after chemical modification. Due to increase in insect breeding worldwide, the amount of this type of waste will be also increasing. Thus, the use of this waste fits particularly well with the principles of the circular economy. In the future, on the basis of our findings, testing the ability for absorption of other ions should be checked. This study provides a good justification for the broad testing of puparia and dead adults as biosorbents for real wastewater.

## Figures and Tables

**Table 1:** Physical characteristics of commercial chitin, chitin obtained from *H. illucens* adults (Ch-A), chitin derived from *H. illucens* puparia (Ch-P), the ground form of *H. illucens* adults (A), the ground form of *H. illucens* puparia (P), high-molecular-weight commercial chitosan (CS-HW), medium-molecular-weight commercial chitosan (CS-MW), and low-molecular-weight commercial chitosan (CS-LW).

Materials	BET Surface Area (m^2^·g^−1^)	Average Pore Diameter (nm)	Pore Volume (cm^3^·g^−1^)
Commercial chitin	5.28	15.8	0.0238
Ch-A	2.15	7.7	0.0068
Ch-P	0.11	22.7	0.0043
A	1.46	12.1	0.0053
P	0.03	41.4	0.0039
CS-HW	0.32	32.8	0.0017
CS-MW	1.21	11.9	0.0038
CS-LW	0.60	34.9	0.0036

**Table 2:** Cerium(III) ions sorption parameters for nonlinear kinetic modelling.

Model	Parameters	Sorbent							
		chitin	Ch-A	Ch-P	A	P	CS-HW	CS-MW	CS-LW
	q_e_	5.20	3.82	4.58	15.06	24.45	25.83	19.49	23.23
PFO	k_1_	0.717	0.310	0.290	0.310	3.232	2.128	0.708	0.853
	q_1_	4.70	3.31	3.89	13.75	24.06	24.52	17.40	21.36
	R^2^	0.903	0.933	0.782	0.902	0.998	0.987	0.949	0.847
	χ^2^	0.222	0.095	0.372	2.038	0.128	0.719	1.580	7.320
PSO	k_2_	0.227	0.132	0.133	0.040	0.087	0.240	0.067	0.054
	q_2_	4.9	3.47	4.02	14.17	24.19	24.87	18.07	22.42
	R^2^	0.966	0.954	0.883	0.950	0.999	0.994	0.978	0.940
	χ^2^	0.078	0.065	0.200	1.041	0.498	0.326	0.670	2.871
Elovich	α	1.88 × 10^3^	18.935	55.600	2.84 × 10^2^	7.47 × 10^22^	9.73 × 10^15^	1.97 × 10^4^	1.93 × 10^2^
	β	2.692	2.536	2.428	0.723	4.321	1.680	0.796	0.628
	R^2^	0.992	0.921	0.980	0.960	1.000	0.998	0.965	0.979
	χ^2^	0.018	0.112	0.034	0.835	0.079	0.106	1.081	1.010

Non-linear kinetic models: pseudo-first-order (PFO), pseudo-second-order (PSO) and Elovich model. Chitin obtained from *H. illucens* adults (Ch-A), chitin derived from *H. illucens* puparia (Ch-P), the ground form of *H. illucens* adults (A), the ground form of *H. illucens* puparia (P), high-molecular-weight commercial chitosan (CS-HW), medium-molecular-weight commercial chitosan (CS-MW), and low-molecular-weight commercial chitosan (CS-LW).

**Table 3:** Cerium(III) ions sorption parameters for nonlinear isotherm modelling.

Model	Parameters	Sorbent							
		chitin	Ch-A	Ch-P	A	P	CS-HW	CS-MW	CS-LW
Langmuir	q_m_	8.08	10.73	12.62	44.93	69.35	37.00	26.48	31.85
	K_L_	0.035	0.011	0.017	0.030	0.225	0.804	0.360	0.797
	R^2^	0.965	0.948	0.985	0.886	0.959	0.903	0.976	0.916
	χ^2^	0.138	0.218	0.115	14.859	20.096	15.040	1.509	9.051
Freundlich	K_F_	1.247	0.447	0.865	5.436	20.856	17.554	10.561	13.998
	N	2.947	1.853	2.109	2.531	3.478	5.582	4.866	5.121
	R^2^	0.874	0.978	0.953	0.821	0.738	0.834	0.901	0.868
	χ^2^	0.494	0.092	0.371	23.326	129.643	25.572	6.199	14.298
Temkin	A	0.398	0.187	0.210	11.640	2.777	47.800	10.572	21.623
	B	1.666	1.890	2.591	4.051	13.305	4.620	3.875	4.404
	R^2^	0.942	0.911	0.963	0.697	0.896	0.901	0.971	0.914
	χ^2^	0.227	0.377	0.291	52.569	51.667	15.229	1.826	9.179

Chitin obtained from *H. illucens* adults (Ch-A), chitin derived from *H. illucens* puparia (Ch-P), the ground form of *H. illucens* adults (A), the ground form of *H. illucens* puparia (P), high-molecular-weight commercial chitosan (CS-HW), medium-molecular-weight commercial chitosan (CS-MW), and low-molecular-weight commercial chitosan (CS-LW).

**Table 4:** Comparison of equilibrium capacities calculated according to the Langmuir model for cerium(III) ions with the literature data.

Sorbent	Equilibrium Capacity (mg∙g^−1^) According to the Langmuir Model	References
Sodium alginate coated magnetite nanoparticles (Alg-Fe_3_O_4_)	33.11	[63]
SBA-15 mesoporous silica	27.67	[64]
Clinoptilolite	30.58	[65]
Chitosan-functionalized magnetite-pectin	9.72	[66]
Magnetite (MNP)	76.92	[67]
*Pinus brutia* leaf powder	17.24	[68]
Magnetic chitosan/yeast	73.53	[69]
Chitin	8.08	This paper
Ch-A	10.73	
Ch-P	12.62	
A	44.93	
P	69.35	
CS-HW	37.00	
CS-MW	26.48	
CS-LW	31.85	

## Data Availability

Data may be provided upon request to the corresponding author.

## Supplementary Materials

The following supporting information can be downloaded at: https://www.mdpi.com/article/10.3390/ma17215255/s1, Figure S1: EDS patterns for (a) commercial chitin, (b) CS-HW, (c) CS-MW, (d) CS-LW.; Figure S2: Nonlinear fitting of kinetic models for the Ce(III) ions sorption on (a) commercial chitin, (b) Ch-I, (c) Ch-W (d) I, (e) W, (f) CS-HW, (g) CS-MW, (h) CS-LW.; Figure S3: Nonlinear fitting of isotherm models for the Ce(III) ions sorption on (a) commercial chitin, (b) Ch-I, (c) Ch-W (d) I, (e) W, (f) CS-HW, (g) CS-MW, (h) CS-LW.; Table S1: Circle Equivalent (CE) diameter distribution of tested materials presented in percentiles sections. CE diameter is the diameter of a circle with the same area as the 2D image of the particle.
