# Supplementary material for: Better Ce (III) Sorption Properties of Unprocessed Chitinous Waste from Hermetia illucens than Commercial Chitosans
Source: Materials (Basel). 2024 Oct 29;17(21):5255. doi: 10.3390/ma17215255 (PMC11547605; doi:10.3390/ma17215255)
Supplement: Supplementary file 1 [file materials-17-05255-s001.zip › materials-3252565-supplementary.pdf]

Better Ce (III) sorption properties of unprocessed chitinous waste from *Hermetia illucens* than commercial chitosans

Justyna Bąk[a], Piotr Bulak[b*], Monika Kaczor[b], Dorota Kołodyńska[a], Andrzej Bieganowski[b]

[a] Department of Inorganic Chemistry, Institute of Chemical Sciences, Faculty of Chemistry, Maria Curie-Skłodowska University, Maria Curie-Skłodowska Sq. 2, 20-031, Lublin, Poland

[b] Institute of Agrophysics, Polish Academy of Sciences, Doświadczalna 4, Lublin 20-290, Poland

Supplementary materials

2.4. Analytical methods

The amount of adsorbed Ce(III) ions (q_t) and the equilibrium capacity (q_e) were calculated according to:

$$q_t = \frac{(C_0 - C_t)V}{m} \quad (1)$$

$$q_e = \frac{(C_0 - C_e)V}{m} \quad (2)$$

where: C_0 (mg/L) - initial concentration, C_t (mg/L) - concentration after time, C_e (mg/L) - equilibrium concentration, m (g) - sorbent mass, V (L) - volume of solution.

The nonlinear form of kinetic models (pseudo firs order - PFO (3), pseudo second order - PSO (4) and Elovich (5)) are used to describe the sorption of Ce(III) ions on biopolymers:

$$q_t = q_e(1 - \exp(-k_1 t)) \quad (3)$$

$$q_t = \frac{k_2 q_e^2 t}{1 + k_2 q_e t} \quad (4)$$

$$q_t = (\frac{1}{\beta})\ln(1 + \alpha\beta t) \quad (5)$$

where: k_1 (1/min) - equilibrium rate constant of PFO model, k_2 (g/mg min) - equilibrium rate constants of PSO one, α (mg/g min) - initial adsorption rate, β (g/mg) - desorption constant which is related to the chemisorption activation energy.

The nonlinear form of isotherm models (Langmuir (6), Freundlich (7), Temkin (8)) are used to describe the sorption of Ce(III) ions on biopolymers:

$$q_e = \frac{q_0 K_L C_e}{1 + K_L C_e} \tag{6}$$

$$q_e = K_F C_e^{1/n} \tag{7}$$

$$q_e = B \ln A + B \ln C_e \tag{8}$$

where: q_0 (mg/g) - monolayer sorption capacity, K_L (L/mg) - characteristic constant for the Langmuir formula related to the affinity for the binding sites, K_F (mg/g) - adsorption capacity characteristics of the Freundlich model, 1/n - Freundlich constant related to the surface heterogeneity, A (L/g) - Temkin constant connected with the maximum binding energy, B (J/mol) - parameter connected with the adsorption heat.

To determine the model that best fits, the experimental data were evaluated using the coefficient of determination (R^2) (9) and chi-square (10) (χ^2) values calculated using Origin software.

$$R^2 = 1 - \frac{\Sigma(q_{e,exp} - q_{e,cal})^2}{\Sigma(q_{e,exp} - q_{e,mean})^2} \tag{9}$$

$$\chi^2 = \Sigma \frac{(q_{e,exp} - q_{e,cal})^2}{q_{e,cal}} \tag{10}$$

where: $q_{e,exp}$ (mg/g) - amount of Ce(III) ions adsorbed at equilibrium determined experimentally, $q_{e,cal}$ (mg/g) - adsorption capacity determined from the kinetic models and $q_{e,mean}$ - mean of $q_{e,exp}$ (mg/g) values.

a)

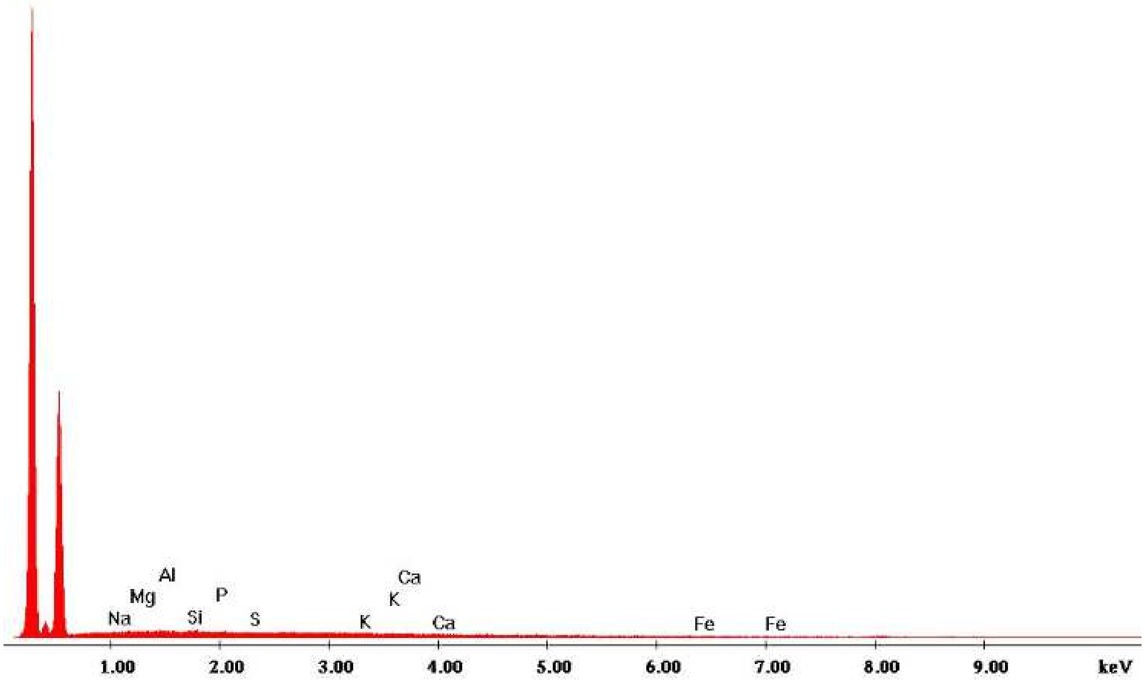
Al
Mg
Na
Si
P
S
Ca
K
K
Ca
Fe
Fe
1.00
2.00
3.00
4.00
5.00
6.00
7.00
8.00
9.00
keV

b)

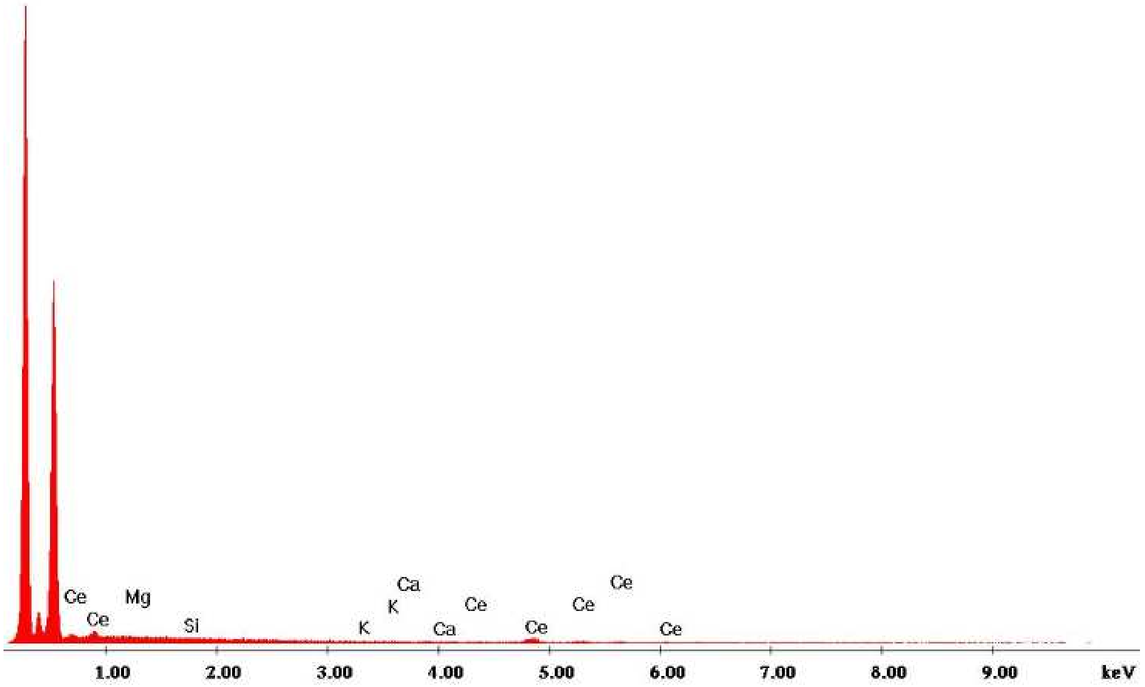
Ce
Ce
Mg
Si
Ca
K
K
Ca
Ce
Ce
Ce
Ce
Ce
1.00
2.00
3.00
4.00
5.00
6.00
7.00
8.00
9.00
keV

c)

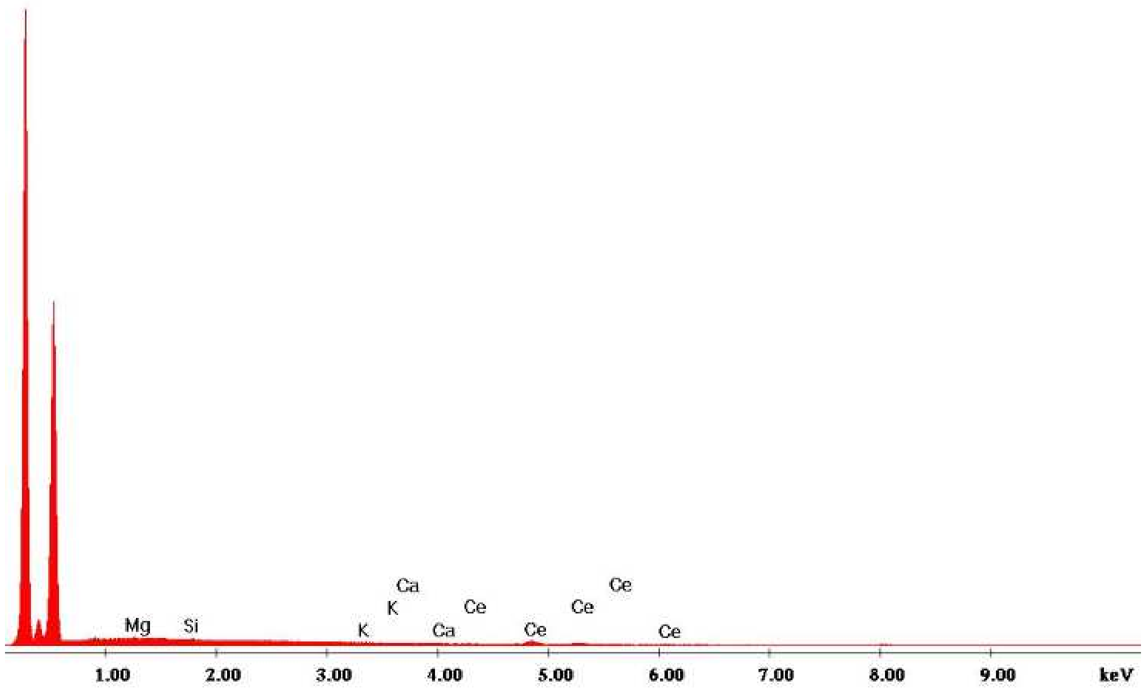

d)

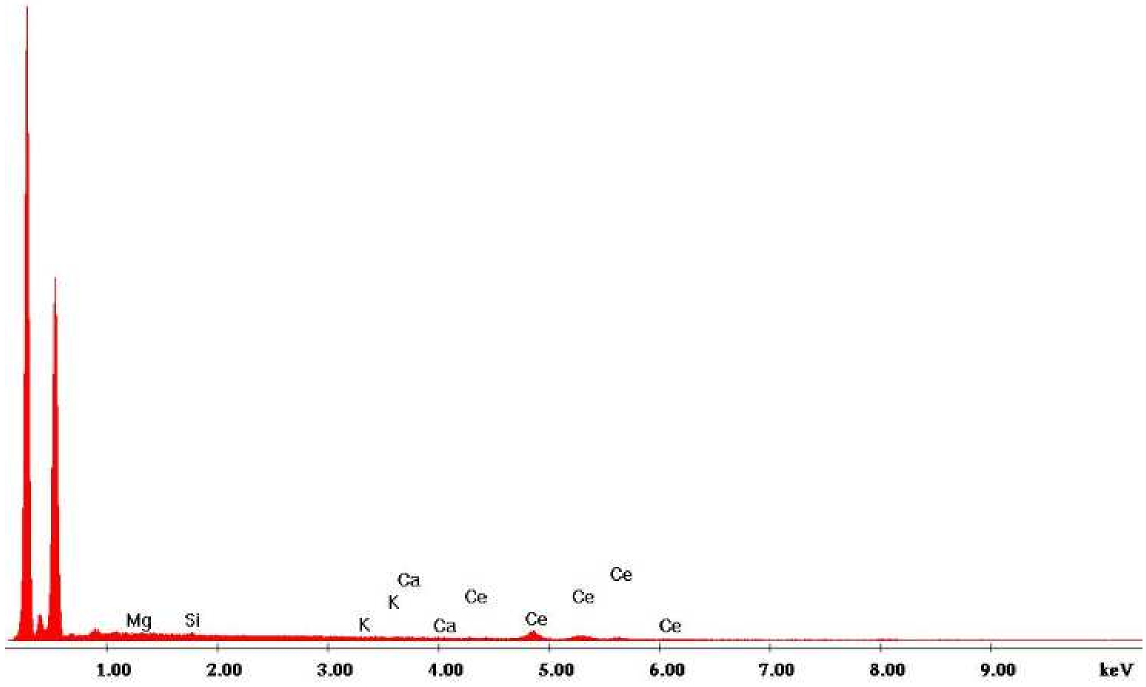

Figure S1. EDS patterns for a) commercial chitin, b) CS-HW, c) CS-MW, d) CS-LW.

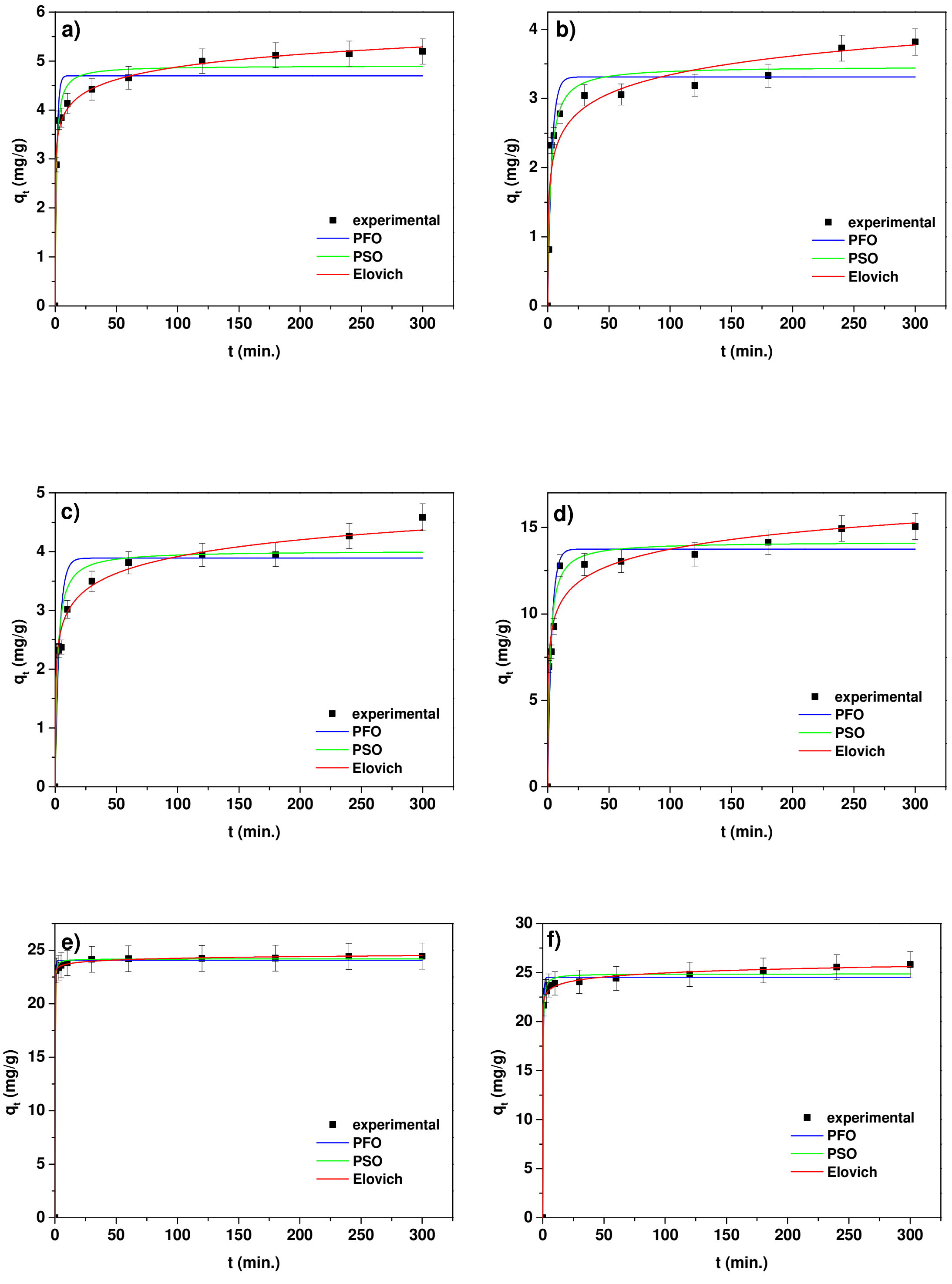
a)
b)
c)
d)
e)
f)
experimental
PFO
PSO
Elovich
q_t (mg/g)
t (min.)

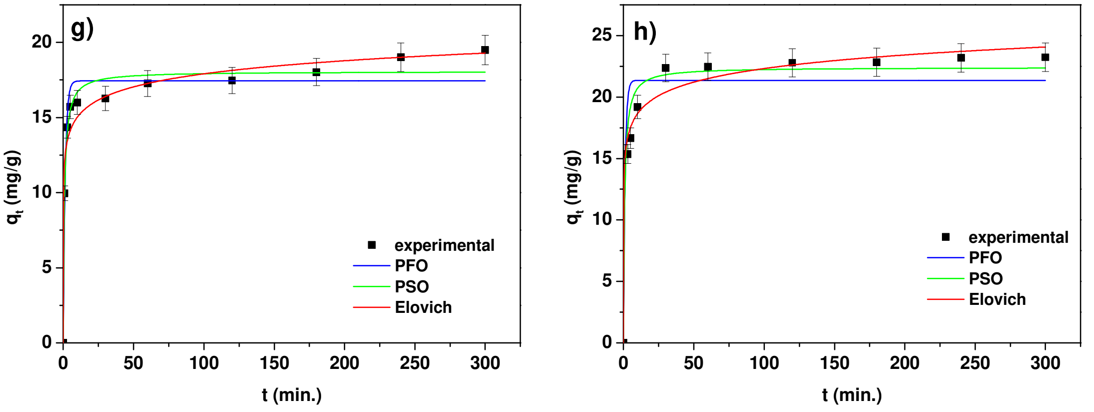

Figure S2. Nonlinear fitting of kinetic models for the Ce(III) ions sorption on a) commercial chitin, b) Ch-I, c) Ch-W d) I, e) W, f) CS-HW, g) CS-MW, h) CS-LW.

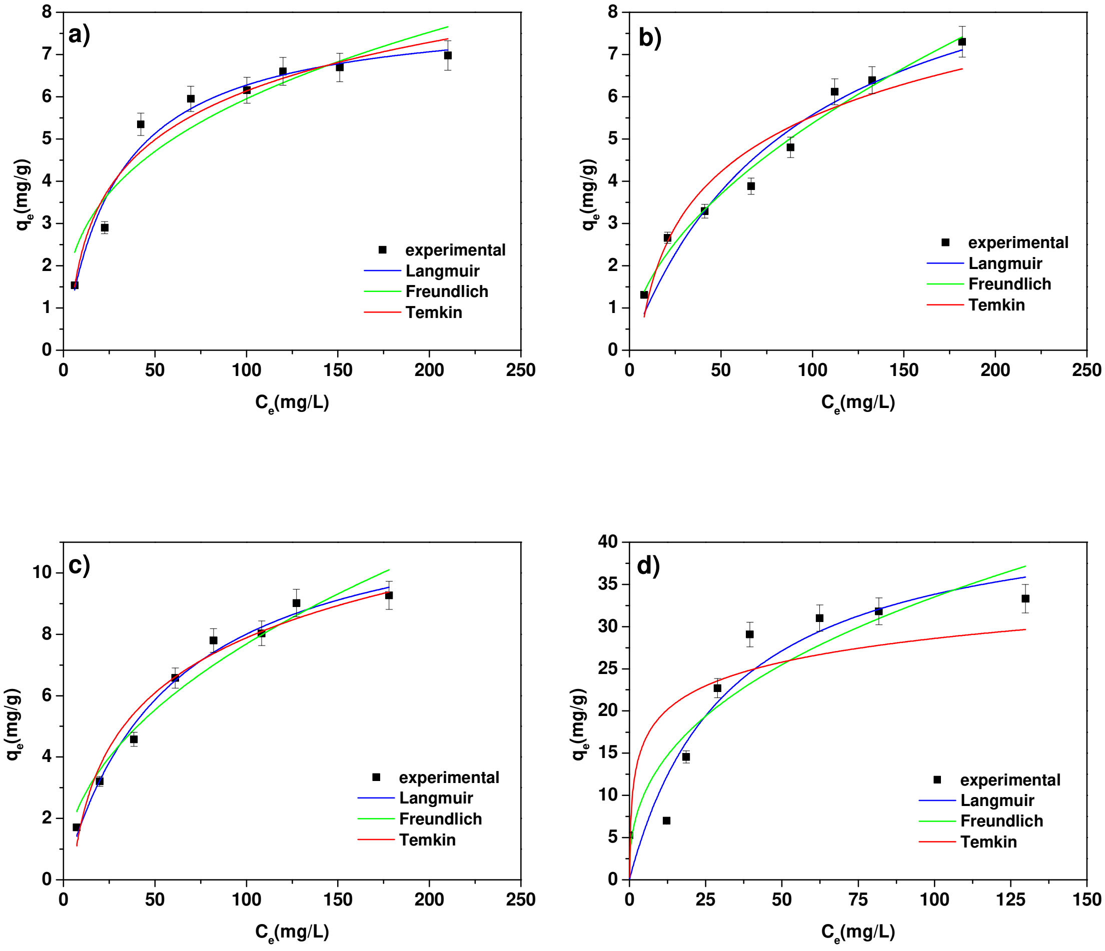

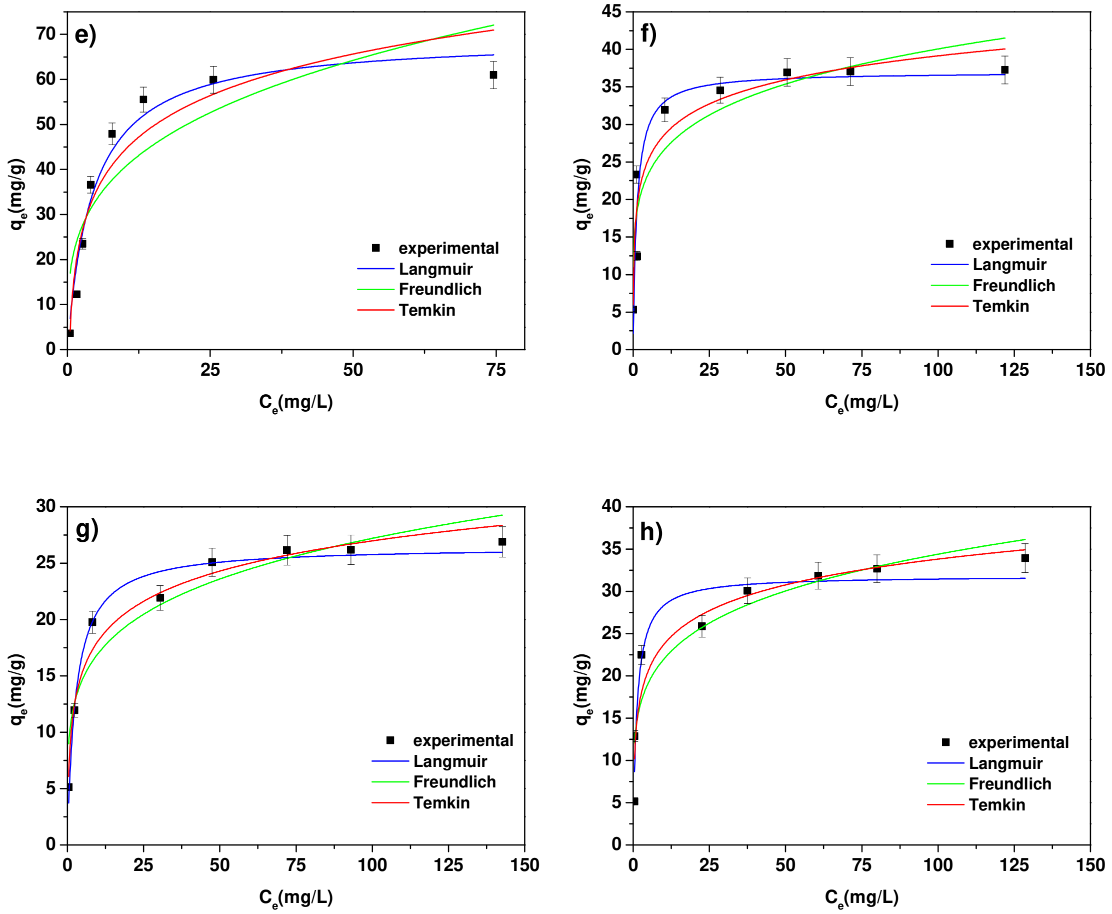

Figure S3. Nonlinear fitting of isotherm models for the Ce(III) ions sorption on a) commercial chitin, b) Ch-I, c) Ch-W d) I, e) W, f) CS-HW, g) CS-MW, h) CS-LW.

Table S1. Circle Equivalent (CE) diameter distribution of tested materials presented in percentiles sections. CE diameter is the diameter of a circle with the same area as the 2D image of the particle.

	**CE Diameter (μm)**		
	**10th percentile**	**50th percentile**	**90th percentile**
Commercial chitin	21.25	33.76	99.72
Commercial chitin Ce	22.66	49.59	167.00
CS-LW	20.93	30.10	80.14
CS-LW Ce	21.81	54.17	167.07
CS-MW	21.27	43.62	238.61
CS-MW Ce	20.56	25.78	270.02
CS-HW	21.19	32.65	103.58
CS-HW Ce	20.81	32.02	394.34
A	21.73	81.38	261.79
A Ce	20.83	29.97	136.80
Ch-A	21.04	34.28	165.14
Ch-A Ce	21.71	40.53	193.57
P	20.72	28.55	65.83
P Ce	20.50	26.45	93.12
Ch-P	20.87	30.36	129.61
Ch-P Ce	21.05	30.18	173.90
